# Pimecrolimus interferes the therapeutic efficacy of human mesenchymal stem cells in atopic dermatitis by regulating NFAT-COX2 signaling

**DOI:** 10.1186/s13287-021-02547-8

**Published:** 2021-08-28

**Authors:** Nari Shin, Namhee Jung, Seung-Eun Lee, Dasom Kong, Nam Gyo Kim, Myung Geun Kook, Hwanhee Park, Soon Won Choi, Seunghee Lee, Kyung-Sun Kang

**Affiliations:** 1 Adult Stem Cell Research Center, Research Institute for Veterinary Science, College of Veterinary Medicine, Seoul National University, 1 Gwanak-ro, Gwanak-gu, Seoul, 08826 Republic of Korea; 2 Stem Cell and Regenerative Bioengineering Institute, Global R&D Center, Kangstem Biotech Co. Ltd., Ace Highend Tower 8, 84, Gasan digital 1-ro, Geumcheon-gu, Seoul, 08590 Republic of Korea

**Keywords:** Atopic dermatitis, Stem cell therapy, Combination therapy, Calcineurin inhibitor, NFAT signaling, COX2-PGE_2_ axis

## Abstract

**Background:**

Human mesenchymal stem cells (hMSCs) therapy has recently been considered a promising treatment for atopic dermatitis (AD) due to their immunomodulation and tissue regeneration ability. In our previous studies, we demonstrated that hMSCs alleviate allergic inflammation in murine AD model by inhibiting the activation of mast cells and B cells. Also our phase I/IIa clinical trial showed clinical efficacy and safety of hMSCs in moderate-to-severe adult AD patients. However, hMSCs therapy against atopic dermatitis have had poor results in clinical field. Therefore, we investigated the reason behind this result. We hypothesized that drug–cell interaction could interfere with the therapeutic efficacy of stem cells, and investigated whether coadministration with pimecrolimus, one of the topical calcineurin inhibitors, could influence the therapeutic potential of human umbilical cord blood mesenchymal stem cells (hUCB-MSCs) in AD.

**Methods:**

hUCB-MSCs were subcutaneously injected to AD-induced mice with or without pimecrolimus topical application. To examine whether pimecrolimus influenced the immunomodulatory activity of hUCB-MSCs, hUCB-MSCs were treated with pimecrolimus.

**Results:**

Pimecrolimus disturbed the therapeutic effect of hUCB-MSCs when they were co-administered in murine AD model. Moreover, the inhibitory functions of hUCB-MSCs against type 2 helper T (Th2) cell differentiation and mast cell activation were also deteriorated by pimecrolimus treatment. Interestingly, we found that pimecrolimus decreased the production of PGE_2_, one of the most critical immunomodulatory factors in hUCB-MSCs. And we demonstrated that pimecrolimus downregulated COX2-PGE_2_ axis by inhibiting nuclear translocation of NFAT3.

**Conclusions:**

Coadministration of pimecrolimus with hMSCs could interfere with the therapeutic efficacy of hMSCs in atopic dermatitis, and this is the first study that figured out the interaction of hMSCs with other drugs in cell therapy of atopic dermatitis. Therefore, this study might give rise to improvement of the clinical application of hMSCs therapy and facilitate the widespread application of hMSCs in clinical field.

**Supplementary Information:**

The online version contains supplementary material available at 10.1186/s13287-021-02547-8.

## Background

Atopic dermatitis (AD) is a chronic and relapsing skin disorder characterized by eczematous skin lesions with severe pruritus and epidermal barrier dysfunction [1]. As the incidence of AD increased, impacting approximately 20% of children and 3% of adults worldwide, it has become a major public health issue [2]. The pathogenesis of AD is characterized by excessive type 2 helper T (Th2) cell-mediated inflammation, resulting in increased serum levels of total IgE and skin barrier dysfunction. IgE-mediated mast cell degranulation induces the release of inflammatory mediators that recruit lymphocytes and eosinophils to skin lesions [3]. The aim of the AD clinical management of is to improve symptoms (pruritus and dermatitis), prevent exacerbations, and minimize therapeutic risks such as bacterial infections. The basic management consists of epidermal barrier repair with emollients and anti-inflammatory drugs such as corticosteroids or calcineurin inhibitors. Calcineurin inhibitors are one of the most common medications prescribed for patients with AD [4–6]. These drugs bind to FK506-binding protein-12 (FKBP-12), inhibit the serine/threonine phosphatase calcineurin, and subsequently prevent nuclear translocation of nuclear factor of activated T cells (NFAT). Consequently, NFAT-mediated transcription of proinflammatory cytokines in T cells is suppressed [7]. However, these classical drugs are insufficient to resolve severe AD or exert significant undesirable side effects [8, 9].

Currently, mesenchymal stem cell (MSC)-based therapy has appeared to be a promising approach to alleviate many diseases, including AD. MSCs are capable of self-renewing and able to differentiate into multiple lineages for damaged tissue regeneration [10]. Additionally, MSCs have been considered as an immunomodulator through the regulation of the proliferation, recruitment and function of innate and adaptive immune cells. In our previous studies, we showed that subcutaneously administered human MSCs (hMSCs) effectively alleviate experimental AD in a mouse model by inhibiting mast cell degranulation through prostaglandin E2 (PGE_2_) and TGF-beta [11]. In the same mouse model of AD, we documented that IV-infused hMSCs significantly reduce disease severity by suppressing B cell proliferation and maturation through cyclooxygenase 2 (COX2) [12]. Furthermore, the development and application of cellular therapies using hMSCs for AD are ongoing, and the hMSC therapy is regarded as a potential candidate for the treatment of AD. We first reported a marked improvement of AD phenotypes of patients who received MSC therapeutics in our phase I/IIa clinical trial [13]. During the clinical trial period, no serious adverse events occurred, and none of the patients discontinued the trial due to adverse events. The results showed the clinical efficacy of human umbilical cord blood-derived mesenchymal stem cells (hUCB-MSCs) in a dose-dependent manner in adult patients with moderate-to-severe AD. In particular, 55% of patients in the high-dose group showed a 50% reduction in the EASI score. The IGA score and SCORAD score in the high-dose group also decreased by 33% and 50%, respectively.

However, in contrast to expectations, most clinical trials using MSCs have produced ambiguous results and these controversial experimental outcomes have been considered a major obstacle towards their widespread clinical application for AD [14]. Therefore, we tried to elucidate the reasons why the different results were obtained. Several limitations of hMSC therapy, such as poor engraftment, survival or donor variation, are suggested as reasons for the discrepancy. Many studies have reported enhancement strategies to resolve these problems, including preconditioning and genetic modification [15–17], although these approaches have yet to be confirmed in the clinic field. Furthermore, a combination strategy might be one of the reasons why the clinical application of hMSCs is complicated. Therefore, a more comprehensive analysis of coadministration with other drugs is needed to establish standardized guidelines for efficient hMSC therapy.

In this study, we hypothesized that the other drugs administered with hMSCs during the clinical trial might have interacted with hMSCs. We investigated whether the combined application of hMSCs and calcineurin inhibitors limits the therapeutic effect of cell therapy in subjects with AD. In the AD mouse model, the combination group showed no or a lower therapeutic effect than the group treated with only hUCB-MSC injection. The coculture of hUCB-MSCs and AD-related immune cells, such as Th2 cells and mast cells, also showed a decreased suppressive effect induced by pimecrolimus. NFAT3 in hUCB-MSCs functioned as a transcription factor of COX2, one of the important factors for the immunomodulation of MSCs, and pimecrolimus may lower the therapeutic effect of hUCB-MSCs by inhibiting the nuclear translocation of NFAT3 in hUCB-MSCs. Here, for the first time, we documented the negative effects of combination therapy of hUCB-MSCs with other medicines and suggested enhancement strategies for the clinical application of hUCB-MSCs.

## Methods

### Mice & induction of AD in NC/Nga mice

**Fig. 1:**
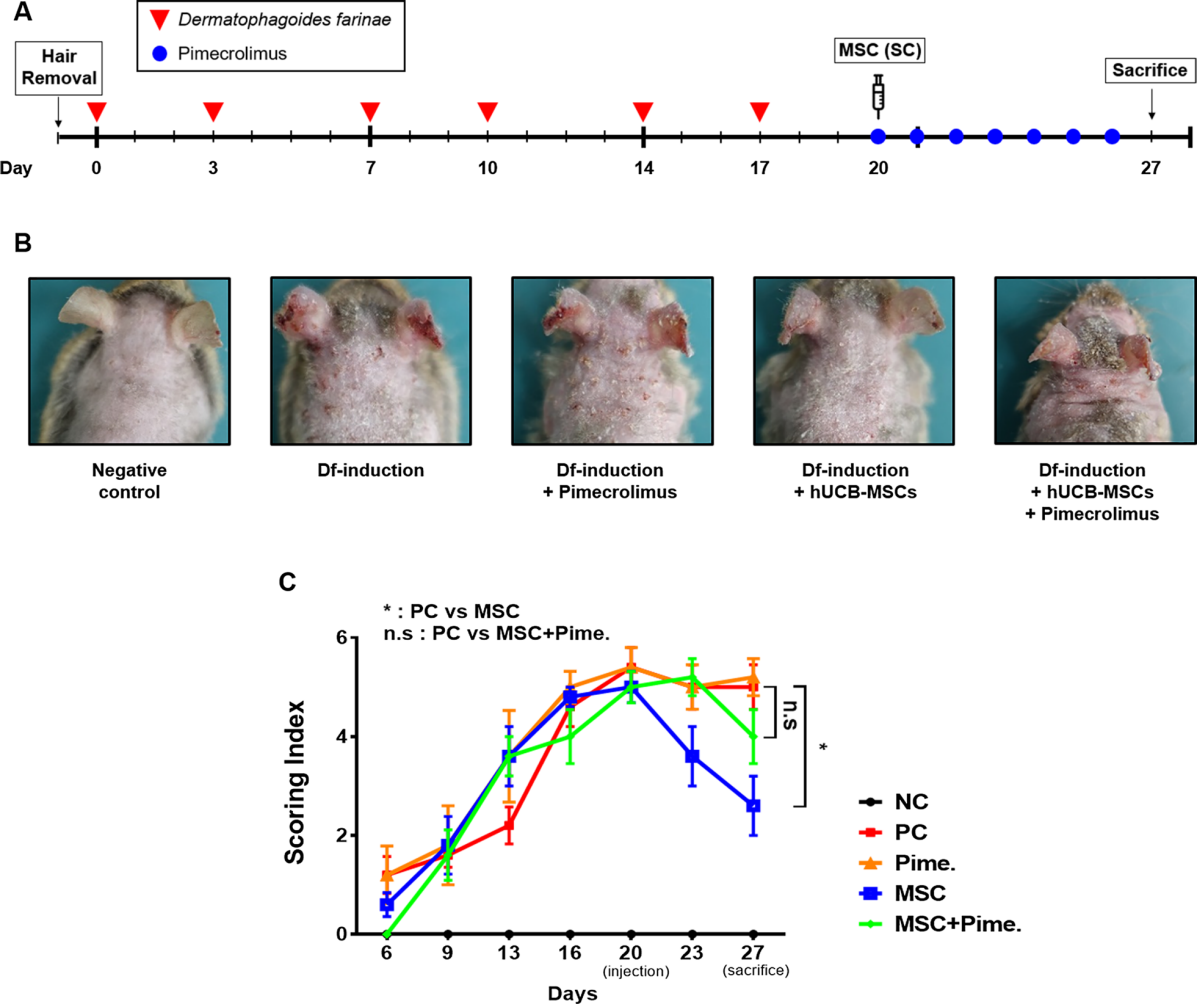
Simultaneous administration of pimecrolimus and hUCB-MSCs deteriorates therapeutic effects of MSCs against atopic dermatitis. AD was induced in NC/Nga mice by repetitive exposure to Df extract in the dorsal and ear regions for 3 weeks. Each group of mice was monitored and sacrificed on day 27 for further analysis. **a** Schematic of the experimental course of AD induction in mice. **b** Representative gross images of dorsal and ear lesions. **c** Clinical severity was assessed by scoring dryness, excoriation, erythema and edema. The Kruskal–Wallis test with Dunn’s post hoc test was used to assess the significance of differences in scoring index at day 27. *N* = 3–5 mice per group. n.s: not significant and *P < 0.05. The results are shown as the mean ± SD

All animal experimental procedures were approved by the Seoul National University Institutional Animal Care and Use Committee (IACUC No. SNU-191122-1-1) in accordance with the guidelines of the committee. NC/Nga mice (male, seven‐week‐old) were purchased from Central Lab Animal Inc. (Seoul, Republic of Korea). Mice were housed in a temperature- and humidity-controlled room in the animal facility of Seoul National University. A total of 23 mice were used. They were divided into groups of 5: NC (Negative control group, *N* = 3), PC (Positive control group/Df-induced group, *N* = 5), Pimecrolimus (Df-induction + Pimecrolimus, *N* = 5), MSC (Df-induction + hUCB-MSCs, N = 5), MSC + Pime. (or hUCB-MSC + Pime, Df-induction + hUCB-MSCs + Pimecrolimus, N = 5). For induction of AD in NC/Nga mice, hair was removed from the lower part of the ear to the upper part of the tail and, NC/Nga mice were left undisturbed for 24 h. Then, 100 mg/head of Df (*Dermatophagoides farinae*; Biostir-AD, Biostir, Kobe, Japan) was applied evenly with a cotton swab to the depilated back and the back of the ear twice a week, for three weeks (total of six times). The test substance was administered on the 3rd day after the last induction (day 20). A total of 2 × 10^6^ hUCB-MSCs were equally subcutaneously administered at five locations of lesions on each mouse. Pimecrolimus (Elidel® cream 1%) was also administered once a day for 7 days from day 20 until day 26. It was applied to the lesion at a dose of 0.2 g/head. Mice were sacrificed on day 27, and blood, ear and dorsal skin were collected. The experimental schedule is shown in Fig. 1a. The severity of the skin lesion was evaluated three times a week one week after the first induction. It was evaluated by the clinical visual evaluation method and symptoms were scored from 0 to 3 points (0: none, 1: mild, 2: moderate and 3: severe). Skin lesion severity was reported as a total score for the following four symptoms: erythema, scarring/dryness, edema, and erosion.

### Histopathological evaluation

Ears and dorsal skin of mice were removed and fixed with 4% paraformaldehyde (Sigma-Aldrich, St. Louis, MO, USA). Fixed tissues were embedded in paraffin. Paraffin‐embedded samples were serially sectioned at a thickness of 5 μm. Sections were stained with hematoxylin and eosin (H&E) or toluidine blue. Slides were observed under a Nikon Eclipse Ti‐U microscope (Nikon, Tokyo, Japan). The epidermal thickness of the dorsal skin and ear was measured in H&E-stained sections, and the number of mast cells was counted in toluidine blue-stained dorsal skin sections.

### Cytokine measurement

Blood was collected from the mice in a BD microtainer tube (BD Bioscience, San Jose, CA, USA) via cardiac puncture and centrifuged at 3500 rpm for 15 min. The supernatant was harvested in a fresh 1.5 ml conical tube. The serum IgE level was measured using a Mouse IgE Uncoated ELISA Kit (Invitrogen, Carlsbad, CA, USA) according to the manufacturer’s instructions.

### Conventional RT-PCR and quantitative real-time PCR

Total RNA was extracted from cultured cells and in vivo tissues using TRIzol reagent (Invitrogen). cDNA synthesis was performed using Superscript™ III reverse transcriptase (Invitrogen). For conventional RT-PCR, the first-strand cDNA was amplified with primer sets for NFAT family member and COX2. The mRNA levels of each gene were normalized using GAPDH as the housekeeping gene. After adequate cycles, aliquots of each sample were electrophoresed on 1.5% agarose gels (w/v) with 1X GelRed (Hayward, CA, USA) to visualize gels under UV light. Quantitative Real-time PCR was performed using SYBR-Green PCR Master Mix with an Applied Biosystems 7500 Real-Time PCR System (Applied Biosystems, Foster City, CA, USA). The Quantitative Real-time PCR reaction was carried out with initial denaturation conditions at 50 °C for 2 min and at 95 °C for 10 min followed by at 95 °C for 15 s and 60 °C for 1 min for 60 cycles. All the reactions were performed in triplicate independently three times and the obtained results were normalized against the GAPDH housekeeping gene. The expression levels of major inflammatory cytokines in lesioned ear skin of mice, TARC and IL-22, were measured for comparative analysis. Also, in vitro experiment, the expression level of TNF in LAD-2 cell, GATA3, STAT6 and IL-13 in Th2 cell and COX2 in hUCB-MSCs were measured. The primer sequences and conditions used in this study are presented in Additional file 4: Table S1.

### Cell culture

hUCB‐MSCs were provided by the Kangstem Biotech GMP Center (Gwangmyeong, Republic of Korea). All experiments using hUCB-MSCs were approved by the IRB of the Public Institutional Bioethics Committee designated by the South Korea Ministry of Health and Welfare (Approval No. P01-201605-BS-02) with informed maternal consent. hUCBs were obtained from 4 donors and total 4 lines of hUCB-MSCs were established. hUCB‐MSCs were cultured in KSB-3 complete media (Kangstem Biotech, Seoul, Republic of Korea) containing 10% fetal bovine serum (Gibco BRL, Grand Island, NY, USA). We used hUCB‐MSCs under passage 6 for all experiments. For mononuclear cell culture and Th2 cell isolation, human peripheral blood mononuclear cells were purchased from Lonza (Lonza, Basel, Switzerland) and they were cultured in RPMI 1640 (Gibco) supplemented with 10% fetal bovine serum. The human mast cell line LAD-2, which was kindly provided by Dr. D. D. Metcalfe of the Center for Cancer Research, National Institutes of Health (Bethesda, MD, USA), was cultured as previously described [12]. Briefly, the cells were cultured in StemPro-34 serum-free medium supplemented with 1X GlutaMAX (Gibco BRL), 100 ng/ml recombinant human stem cell factor (rhSCF, Peprotech, Rock Hill, NJ, USA) and antibiotics. In in vitro culture experiment, hUCB-MSC + Pime group means the cells that cocultured with hUCB-MSCs and pimecrolimus.

### MTT assay

hUCB‐MSCs were seeded at a density of 3 × 10^4^ cells per well in a 24-well cell culture plate. After 24 h of incubation, hUCB‐MSCs were treated with serial tenfold dilutions of pimecrolimus (Sigma-Aldrich, 0.1 ng/ml – 10 μg/ml) for 72 h. After treatment, cells were incubated with 500 μl of MTT solution (0.5 mg/ml) for 4 h. After incubation, MTT solution was removed and 500 μl of DMSO was added to dissolve insoluble formazan crystal. Then, the plate was read immediately and the absorbance of each well was measured at wavelength of 570 nm using an Infinite200 PRO microplate reader (Tecan, Maennedorf, Switzerland).

### Cell cycle assay

After hUCB‐MSCs were treated with pimecrolimus (0.01 ng/ml, 1 ng/ml, 100 ng/ml) for 3 days, cells were harvested and washed with PBS twice and fixed with ice-cold 70% ethanol at -20℃ for over 30 min. Fixed cells were washed with PBS and suspended in 400 μl PBS, containing RNase A (final concentration 6.25 μg/ml) and Propidium iodide (final concentration 50 μg/ml). After incubation in 37℃ for 30 min, cell cycle analysis was performed using a FACSCalibur flow cytometer and Cell Quest software (BD Bioscience).

### Flow cytometry

To assess the effect on hUCB-MSCs, hUCB-MSCs were treated with 100 ng/ml pimecrolimus for 3 days. After treatment, trypsinized cells were incubated with a fluorochrome-conjugated antibody and analyzed. The following antibodies were used: FITC Mouse Anti-Human CD45, PE Mouse Anti-Human HLA-DR, FITC Mouse anti-Human CD105, APC Mouse anti-Human CD73, APC Mouse Anti-Human CD29, FITC Mouse Anti-Human CD44, PE Mouse Anti-Human CD36 and PE Mouse Anti-Human CD34. Whole antibodies were purchased from BD Bioscience. Fluorescence was detected with a FACScalibur flow cytometer and evaluated using Cell Quest software (BD Biosciences).

### Measurement of the secretion of IDO-1 from hUCB-MSCs

hUCB-MSCs were seeded in a 6-well cell culture plate at a density of 1.5 × 10^5^ cells per well. After 24 h of incubation, we treated IFN-γ (20 ng/ml) to hUCB-MSCs for 72 h with or without pimecrolimus (0.01 ng/ml, 1 ng/ml, 100 ng/ml) to detect the secretion of IDO-1. Cell lysates were extracted with cell lysis buffer. Human IDO1/IDO ELISA Kit (LsBio, Seattle, WA, USA) was used for measurement of IDO-1 concentration following the manufacturer's instruction.

### Measurement of the secretion of PGE_2_ and TGF-beta from hUCB-MSCs

Secretion or production of PGE_2_ and TGF-beta from hUCB-MSCs was quantified using ELISA kits in accordance with the manufacturer’s instructions. hUCB-MSCs were seeded in a 6-well cell culture plate at a density of 1.5 × 10^5^ cells per well. After 24 h of incubation, hUCB‐MSCs were treated with pimecrolimus (0.01 ng/ml, 1 ng/ml, 100 ng/ml) for 72 h. The media was collected and centrifuged at 2500 rpm for 5 min to eliminate cell debris. The concentrations were determined using Human TGF‐β1 Quantikine ELISA (R&D Systems, Minneapolis, MN, USA) and Human PGE_2_ Quantikine ELISA (R&D Systems).

### Cell proliferation assay

A total 3 independent cell proliferation assay experiments were conducted. (1) To assess the effect of pimecrolimus against the proliferation of hUCB-MSCs, hUCB-MSCs were seeded at a density of 1.5 × 10^4^ and pimecrolimus (0.01 ng/ml, 1 ng/ml, 100 ng/ml) was treated for 72 h after attachment of cells. (2) To assess the suppression ability of hUCB-MSCs to mononuclear cell proliferation, we conducted coculture experiment of hUCB-MSCs and mononuclear cell. hUCB-MSCs were seeded at a density of 1.5 × 10^4^ (1:10) per well in a 96-well cell culture plate. After attachment of hUCB-MSCs, 1.5 × 10^5^ human peripheral blood mononuclear cells (hPBMCs, Lonza) were seeded per well with ConA (5 μg/ml) and cultured for 5 days. During 5 days, hPBMCs were treated with pimecrolimus (100 ng/ml) with or without hUCB-MSCs. (3) To assess the suppression ability of hUCB-MSCs to mast cell proliferation, we conducted coculture experiment of hUCB-MSCs and LAD-2 cells. hUCB-MSCs were seeded at a density of 1.5 × 10^4^ (1:10) per well in a 96-well cell culture plate. After attachment of hUCB-MSCs, 1.5 × 10^5^ LAD-2 were seeded per well and cultured for 24 h. Proliferation was detected by a bromodeoxyuridine (BrdU) ELISA kit (Roche, Indianapolis, IN, USA). After culture, BrdU labeling solution (100 μM) was added. The culture medium containing BrdU solution was removed after overnight incubation at 37 °C, and the cells were fixed with FixDenat solution for 30 min at room temperature. After fixation the cells were incubated with an anti-BrdU-POD working solution for 90 min at room temperature. Following three washing steps, a substrate solution was added to the cells and incubated for 5–30 min at room temperature. After sufficient color development, the OD values were read at 450 nm on a microplate reader (TECAN, Zürich, Switzerland). Within the coculture experiment, we have calibrated the proliferation of hPBMCs and LAD-2 cells by subtracting OD values of hUCB-MSCs cultured wells from OD values of cocultured wells.

### Th2 and hUCB-MSC coculture

We conducted a coculture experiment with hUCB-MSCs and Th2 cells to assess inhibitory effect of hUCB-MSCs on Th2 cell differentiation. hUCB-MSCs (3 × 10^4^) were seeded on a Transwell membrane (pore size 8 μm; Corning, Bedford, MA, USA). CD4^+^ T cells were purified from human peripheral blood mononuclear cells (Lonza) using a human CD4^+^ T cell isolation kit (Miltenyi Biotec, Bisley, UK) according to the manufacturer’s instructions. After the attachment of hUCB-MSCs, 5 × 10^5^ CD4^+^ T cells were cultured in the well beneath the insert for 5 days. Cells were cultured with RPMI 1640 medium containing 10% heat-inactivated fetal bovine serum, Dynabeads Human T-Activator CD3/CD28 (Gibco BRL), IL-2 (20 ng/ml), IL-4 (20 ng/ml) and IL-6 (20 ng/ml) to induce Th2 differentiation. All recombinant human cytokines were purchased from Peprotech. For 5 days, CD4^+^ T cells were treated with pimecrolimus (100 ng/ml) at the indicated concentrations with or without hUCB-MSCs. After 5 days of coculture, Th2 differentiation was analyzed by detecting the levels of the CD4 (FITC mouse anti-human CD4; BD Biosciences) and IL-4 (PE mouse anti-human IL-4; BD Biosciences) proteins using a FACSCalibur flow cytometer and Cell Quest software (BD Biosciences).

### Beta-hexosaminidase assay

hUCB-MSCs (1.5 × 10^5^ per well) were seeded in 24-well cell culture plates and incubated for 24 h. After incubation, 1.5 × 10^5^ LAD-2 human mast cells were added and sensitized with 100 ng/ml human myeloma IgE (Merck-Millipore, Burlington, MA, USA) for 24 h with or without pimecrolimus (100 ng/ml). The next day, LAD-2 cells were washed and challenged with 3 μg/ml goat anti-IgE (polyclonal antibody, Merck-Millipore) for 1 h. After challenge, the induction of mast cell degranulation was stopped on ice, and supernatants were harvested after centrifugation. The supernatant (60 μl) was transferred to a 96‐well plate and mixed with an equal volume (60 μl) of 7.5 mM ρ‐nitrophenyl‐Ν‐acetyl‐β‐d‐glucosaminide substrate solution (dissolved in 0.05 M citric acid, pH 4.5, Sigma-Aldrich). The mixture was incubated in a shaking incubator (120 rpm) for 2 h at 37 °C, and then 120 μl of 0.2 M glycine (pH 10.7) was added. The absorbance at 410 nm was measured using a microplate reader (TECAN). The release of β‐hexosaminidase was calculated as the percentage of the total β‐hexosaminidase content in stimulated mast cells, as determined by lysing cells in buffer containing 0.1% Triton X‐100.

### Western blotting

Whole-cell lysates were prepared with the protein lysis buffer Pro-prep (Intron biotechnology, Gyeonggi, Korea), and nuclear and cytosolic fractionation was conducted as described previously [18]. The concentration of protein was measured using a Bio-Rad DC protein assay kit (Bio-Rad Laboratories, Hercules, CA, USA), with bovine serum albumin (BSA) as the standard. For each protein sample, a 10 μg aliquot was separated by 8% or 10% sodium dodecyl sulfate polyacrylamide gel electrophoresis (SDS-PAGE) and transferred to a nitrocellulose membrane. After blocking with 3% BSA in Tris-buffered saline (TBS-T), the blots were probed overnight at 4 °C with the following primary antibodies: COX2 (Cell Signaling Technology, Beverly, MA, USA, 12282, 1:1000), NFAT3 (Santa Cruz Biotechnology, Santa Cruz, CA, USA, sc-271127, 1:200), p-NFAT3 (Santa Cruz Biotechnology, sc-135771, 1:200), Lamin A/C (Santa Cruz Biotechnology, sc-7292, 1:200), Alpha tubulin (Abcam, ab18251, 1:1000), GAPDH (Merck-Millipore, MAB374, 1:2000). After washing the membranes with TBS-T (three times, 5 min each), the membranes were blotted with secondary antibodies at room temperature for 1 h. The proteins were detected with enhanced chemiluminescence (ECL) reagent (GE Healthcare Life Science, Buckinghamshire, UK).

### Immunocytochemistry staining

After the treatments indicated in each figure legend, hUCB-MSCs were fixed with 4% paraformaldehyde at room temperature for 10 min, permeabilized by 0.05% Triton X-100 solution for 10 min and blocked with 5% normal goat serum for 1 h. The cells were subsequently stained with specific primary antibodies against COX2 (Abcam, Cambridge, MA, USA, ab15191, 1:100) and NFAT3 (Santa Cruz Biotechnology, 1:50) at 4℃ for overnight. The cells were then incubated with Alexa 488-labeled secondary antibodies (Molecular Probes, Eugene, OR, USA; 1:1000) for 1 h at room temperature. For counterstaining, nuclei were stained with DAPI. The images were captured by a confocal microscope (Nikon, Eclipse TE200).

### Statistical analysis

The mean values of all data are presented as the means ± SD. The significance of differences in the data was determined using Kruskal–Wallis test with Dunn’s post hoc test for multiple groups and is indicated in the figure legends. Statistical analyses were performed using GraphPad Prism version 7.0 (GraphPad Software, San Diego, CA, USA).

## Results

### Combined administration of hUCB-MSCs with pimecrolimus adversely affects the therapeutic potential of hUCB-MSCs against AD mouse model

As a calcineurin inhibitor, pimecrolimus is one of the most common anti-inflammatory drugs used to treat AD. We first investigated whether pimecrolimus influences the therapeutic effect of hUCB-MSCs on AD. As for AD mouse model, we used a Df-induced murine AD model. Df extract was repeatedly applied onto the dorsal surface and ear of NC/Nga mice twice a week for three weeks (Fig. 1a). As mentioned in our previous studies [12, 16], 2.0 × 10^6^ hUCB-MSCs were subcutaneously injected per head on day 20 when AD was fully induced. Pimecrolimus was topically administered once a day from day 20 until sacrifice. Compared to the negative control group, the Df-induced group exhibited itching, erythema, and hemorrhage on the dorsal skin and ear, followed by edema, excoriation, erosion, scaling, and dryness of the skin (Fig. 1b). The affected area was alleviated in the hUCB-MSC-injected group, but the hUCB-MSC + Pime group were slightly improved. For quantification, we regularly evaluated the clinical severity until day 27 by scoring the symptoms: erythema, scarring/dryness, edema, and erosion (Fig. 1c). hUCB-MSC-injected groups exhibited decrease in clinical severities after injection. On day 27, Df-induced mice showed moderate dermatitis scores of 5 ± 0.96, and the hUCB-MSC-injected group showed decreased scores of 2.6 ± 1.26, or 48% lower than the Df-induced group. However, the hUCB-MSC + Pime group scored 4 ± 0.96, confirming that coadministration did not significantly alleviate the clinical severity compared to the hUCB-MSC-injected group. These visual observations indicated that coadministration with pimecrolimus hindered the therapeutic effect of hUCB-MSCs on a murine AD model.

### Pimecrolimus interrupts the therapeutic potential of hUCB-MSCs based on histological and molecular analyses of Df-induced AD in mice

**Fig. 2:**
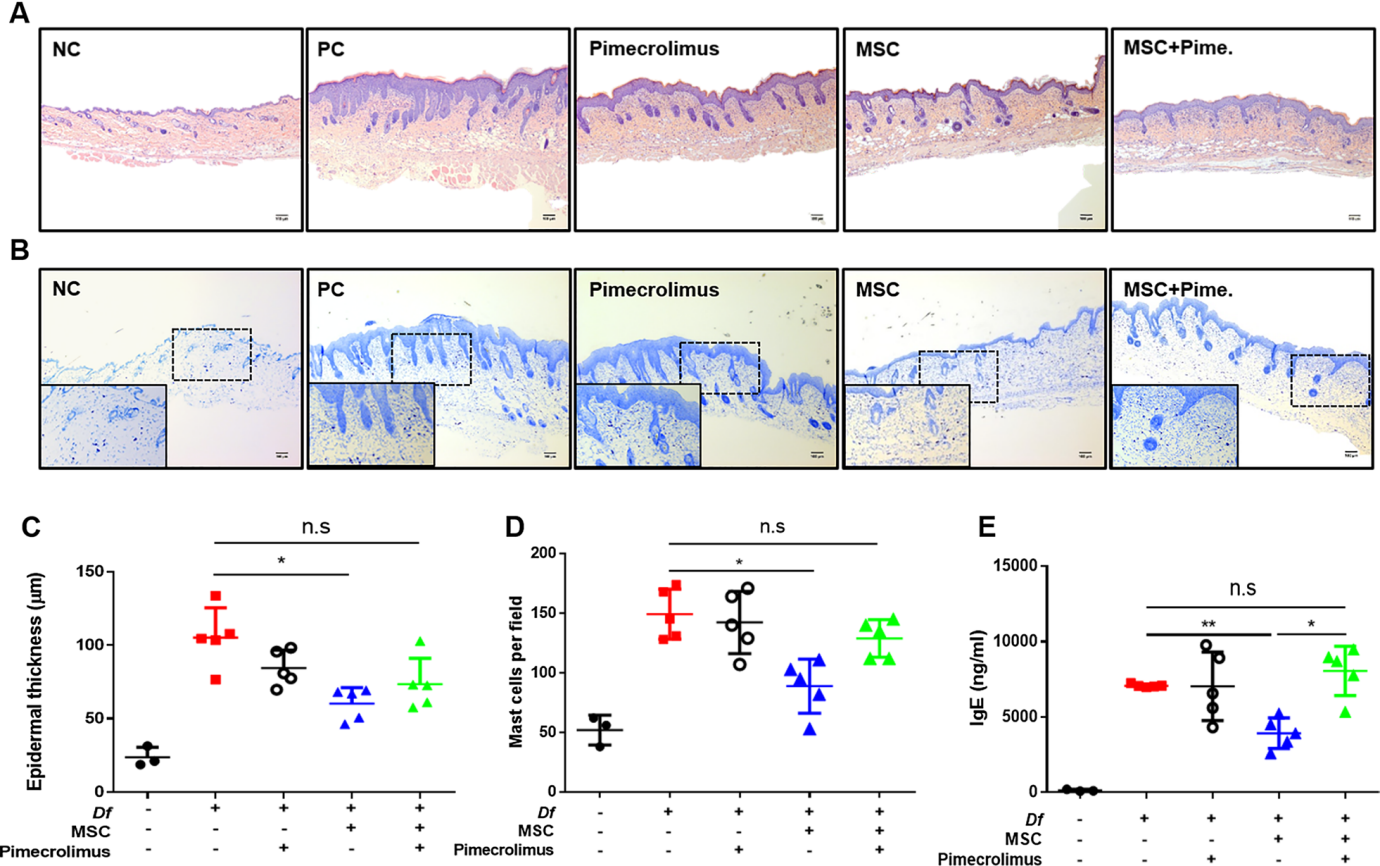
Coadministration of pimecrolimus and hUCB-MSCs inhibits the regulatory effect of hUCB-MSCs on skin inflammation in Df-induced AD mice. **a**–**d** Experimental AD was induced in mice, and skin samples were collected on day 27 for further ex vivo examinations. **a** Representative images of H&E-stained sections, Bar = 100 μm. **b** Representative images of toluidine blue-stained sections, Bar = 100 μm. **c** Epidermal thickness of the dorsal skin was measured in H&E-stained sections. **d** The number of mast cells was measured in toluidine blue-stained sections. **e** The levels of IgE in mouse sera were determined using an ELISA. NC: negative control group, PC: positive control group (Df-induced group), Pimecrolimus: Df-induction + pimecrolimus, MSC: Df-induction + hUCB-MSCs, MSC + Pime.: Df-induction + hUCB-MSCs + Pimecrolimus. *N* = 3–5 mice per group. The Kruskal–Wallis test with Dunn’s post hoc test was used to compare other groups with Df-induced group **c**–**e** n.s: not significant, *P < 0.05 and **P < 0.01. The results are presented as the means ± SD

We have further examined the skin; both dorsal and ear, and serum of AD mice on day 27. H&E-stained dorsal skin samples were investigated to analyze the histopathological changes (Fig. 2a). We observed histological pathologies of experimental AD, including edema and epidermal hyperplasia and lymphocyte infiltration, in Df-induced mice. AD pathologies were attenuated in the hUCB-MSC-injected group. However, little attenuation of epidermal pathology was observed in the hUCB-MSC + Pime group compared with the hUCB-MSCs-injected group. Additionally, the ear skin also presented the representative histology of AD induced by Df, and it displayed a similar pattern to the dorsal skin analysis (Additional file 1: Figure S1A). Furthermore, the thickness of the epidermis in H&E-stained dorsal skin and ear skin was measured (Fig. 2c and Additional file 1: S1B). The hUCB-MSC injection resulted in a significant decrease of 42.7% in dorsal epidermal thickness, whereas the hUCB-MSC + Pime group did not differ from the Df-induced group (Fig. 2C). The epidermal thickness of ear skin also showed a similar trend to the analysis of dorsal skin (Figure Additional file 1: S1B). We next evaluated the number of mast cells in the dorsal skin of mice using toluidine blue staining (Fig. 2b). Based on toluidine blue-positive cell counts, the hUCB-MSC injection significantly suppressed the infiltration of mast cells, while coadministration with pimecrolimus countered the inhibitory effects of hUCB-MSCs on mast cell infiltration (Fig. 2d). We further analyzed the gene expression levels of chemokines and cytokines in the ear skin lesions of the mice to investigate whether pimecrolimus altered the immune response (Additional file 1: Figures S1C and S1D). TARC and IL-22 are related to skin inflammation, and their mRNAs are expressed at high levels in AD lesions. The single hUCB-MSC treatment or pimecrolimus treatment significantly decreased the expression levels of TARC and IL-22 mRNAs. However, both TARC and IL-22 levels in the hUCB-MSC + Pime group returned to the levels of those in the Df-induced AD mouse group. We also measured the serum IgE level, one of the major hallmarks of AD [19] (Fig. 2e). The serum IgE level was significantly decreased by 45% in the hUCB-MSC injection group, but the serum IgE level of the hUCB-MSC + Pime group was similar to that of Df-induced group. Taken together, the hUCB-MSC injection significantly reduced the clinical severity of AD in mice, but coadministration of pimecrolimus suppressed the therapeutic effects of hUCB-MSCs on a murine AD model.

### Pimecrolimus has no effect on the cytotoxicity or marker expression of hUCB-MSCs

We first investigated the change in MSC properties induced by pimecrolimus to identify the reason why therapeutic effect of hUCB-MSCs is suppressed by pimecrolimus. hUCB-MSCs were treated with pimecrolimus for 72 h to examine the effect of pimecrolimus on the viability of hUCB-MSCs using MTT assay. A range of 0.1 ng/ml–1 μg/ml pimecrolimus did not induce significant difference in viability compared with the control group (Additional file 2: Figure S2A). Additionally, the administration of pimecrolimus at concentrations ranging from 0.01 ng/ml–100 ng/ml did not cause any changes in the proliferative capacity (Additional file 2: Figure S2B), cell cycle profile (Additional file 2: Figure S2C), or cellular morphology (Additional file 2: Figure S2D). In addition, these treatments did not alter the expression levels of three positive and three negative MSC markers; CD45, CD34, CD36, CD105, CD29 and CD73 (Additional file 2: Figure S2E). These findings suggested that pimecrolimus did not alter the cellular morphology and biological characteristics of hUCB-MSCs.

### Pimecrolimus exerts a suppressive effect on the immunomodulatory functions of hUCB-MSCs

**Fig. 3:**
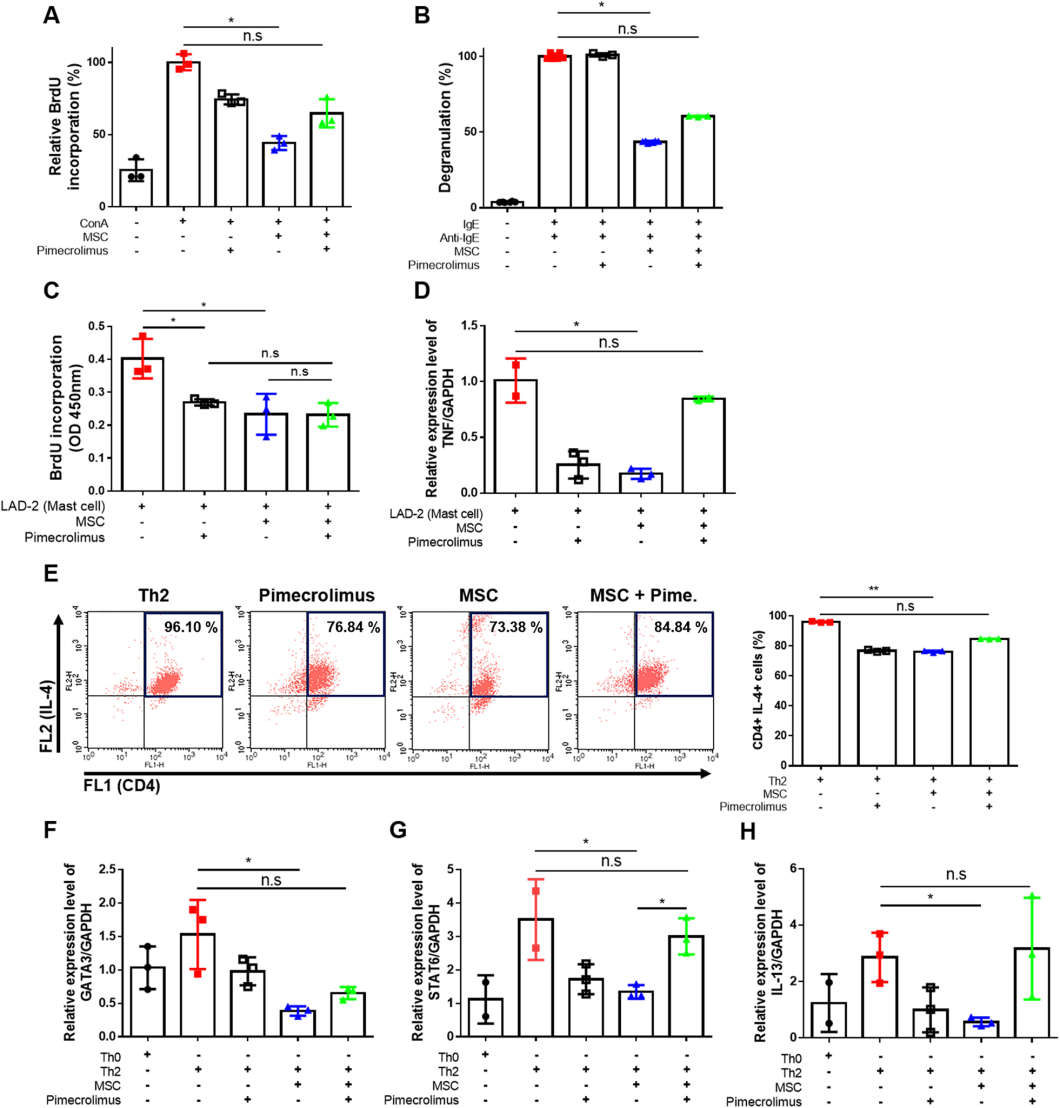
Pimecrolimus exerts a suppressive effect on the immunomodulatory functions of hUCB-MSCs in vitro. Cells were treated with 100 ng/ml pimecrolimus during culture. **a** The immunosuppressive properties of pimecrolimus-treated hUCB-MSCs were determined using the MLR assay by measurement of co-cultured hPBMCs’ proliferation using the BrdU assay. **b** After coculture with hUCB-MSCs during the sensitization period (24 h), LAD-2 cells were challenged with anti-IgE. The degranulation rate of LAD-2 cells was assessed by detecting β-hexosaminidase in the culture medium. **c** The proliferation rate of LAD-2 cells was assessed using the BrdU assay.  **d** Expression levels of TNF mRNA in LAD-2 cells were determined using Quantitative Real-time PCR. **e** The expression of CD4 and IL-4 was determined using a flow cytometry analysis. Th2: Th2 cells, Pimecrolimus: Th2 cells treated with pimecrolimus, MSC: Th2 cells cocultured with MSCs, MSC + Pime.: Th2 cells cocultured with MSCs and pimecrolimus. **f**–**h** Expression levels of the GATA3 (**f**), STAT6 (**g**) and IL-13 (**h**) mRNAs in Th2 cells were determined using Quantitative Real-time PCR. In vitro experiments were performed in triplicate using hUCB-MSCs isolated from each donor (*N* = 3). All experiments shown in this figure were analyzed using the Kruskal–Wallis test with Dunn’s post hoc test. n.s: not significant, *P < 0.05 and **P < 0.01. The results are presented as the means ± SD

As described above, we determined that coadministration with pimecrolimus decreased the therapeutic effect of the hUCB-MSC treatment, but pimecrolimus was not cytotoxic to hUCB-MSCs. Thus, we next examined whether pimecrolimus influenced the immunomodulatory activity of hUCB-MSCs. hPBMCs were cocultured with hUCB-MSCs to evaluate the general immunosuppressive property (Fig. 3a). The ConA-induced proliferation of hPBMCs was significantly inhibited when they were cocultured with hUCB-MSCs or treated with pimecrolimus. However, when hPBMCs were coadministered with pimecrolimus and hUCB-MSCs simultaneously, there was no change in the proliferation of hPBMCs compared with control group.

Mast cells are important effector cells in allergic responses, and an increased number of mast cells are detected in AD skin lesions [20]. Therefore, we investigated whether treatment with pimecrolimus inhibited the suppressive effect of hUCB-MSCs on the function of mast cells. We cocultured the human mast cell line LAD-2 and hUCB-MSCs with pimecrolimus (Fig. 3b–d). hUCB-MSCs significantly suppressed the degranulation of mast cells, but coadministration with pimecrolimus reduced this effect (Fig. 3b). The proliferation of mast cells was decreased in the pimecrolimus-treated group and hUCB-MSC coculture group, but the hUCB-MSC + Pime group did not show a synergistic effect on the proliferation of mast cells (Fig. 3c). hMSCs are also known to suppress the production of proinflammatory cytokines, such as TNF-α, in mast cells [21]. hUCB-MSC coculture suppressed the expression of the TNF-α mRNA, but no suppressive effect was observed in the pimecrolimus-treated coculture group (Fig. 3d). Based on these results, coadministration of pimecrolimus, when cocultured with mast cells, limited the inhibitory effect of hUCB-MSCs on mast cells.

As previously reported, Th2 cells predominate in acute AD and produce increased levels of related cytokines, including IL-4 and IL-13, which induce the production of IgE by B cells [22]. Therefore, we investigated the immunomodulatory effects of hUCB-MSCs on lymphocyte differentiation and function. We discovered that hUCB-MSCs and pimecrolimus decreased the differentiation rate of CD4^+^ IL-4^+^ Th2 cells, whereas the rate of differentiation to Th2 cells in the hUCB-MSC + Pime group with pimecrolimus was as high as that in the Th2 control group (Fig. 3e). We also analyzed the gene expression levels in Th2 cells after differentiation. Coculture with hUCB-MSCs decreased the expression level of GATA3, the key transcription factor for Th2 cell differentiation, and STAT6, which induces the expression of GATA3 [23, 24]. However, pimecrolimus inhibited down-regulation of the expression of both transcription factors induced by hUCB-MSCs (Fig. 3f and g). Additionally, the mRNA expression level of IL-13, one of the most important Th2-type cytokines, was decreased in the hUCB-MSC coculture group. However, coadministration of hUCB-MSCs and pimecrolimus did not change the IL-13 mRNA expression level in Th2 cells (Fig. 3h). In summary, we determined that the pimecrolimus disturbed the immunomodulatory effect of hUCB-MSCs on cells in the AD environment, including Th2 cells and mast cells.

### Pimecrolimus reduces PGE_2_ production by inhibiting COX2 expression in hUCB-MSCs

**Fig. 4:**
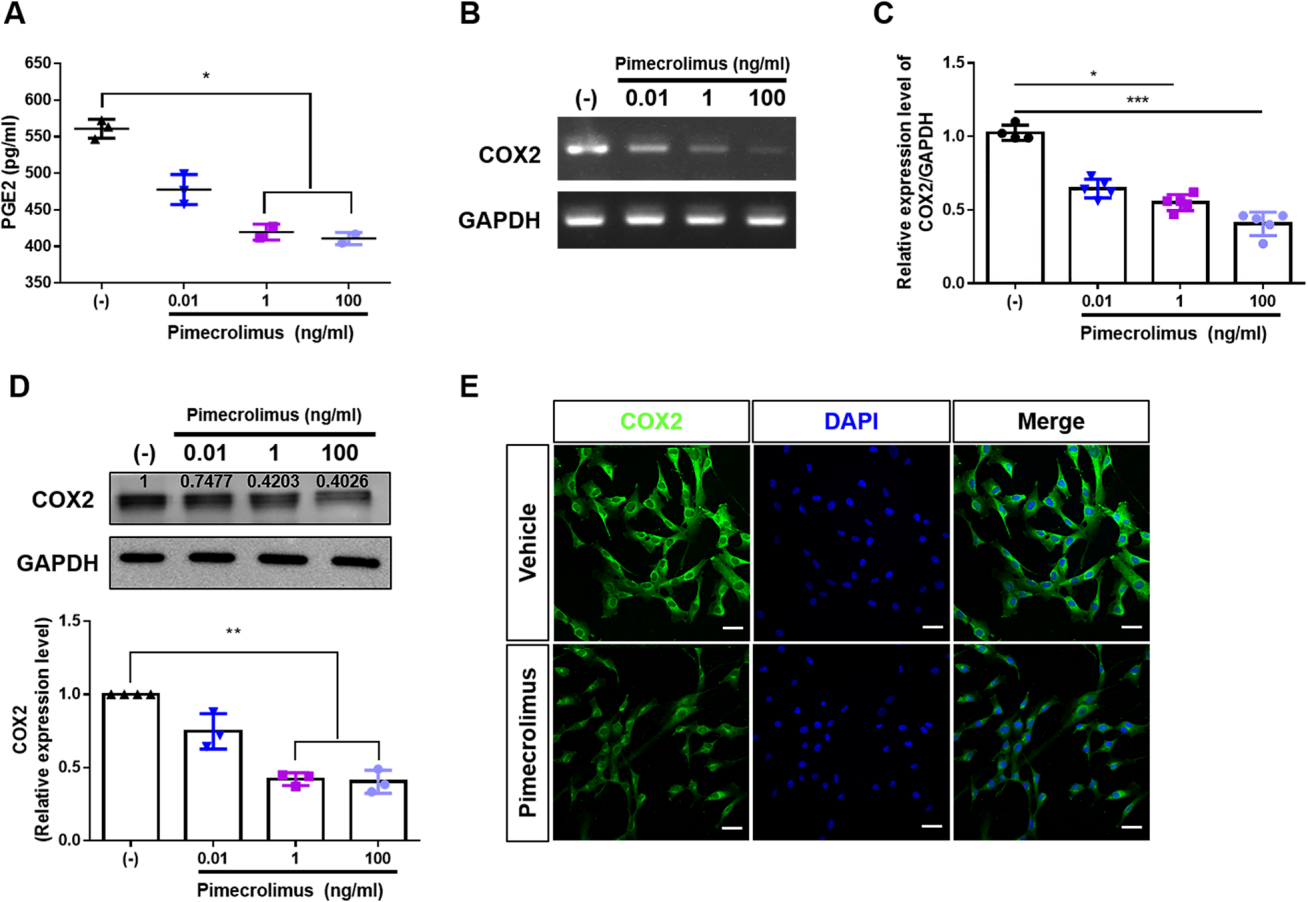
Pimecrolimus reduces PGE_2_ production by regulating COX2 expression in hUCB-MSCs. hUCB-MSCs were treated with **a**–**d** the indicated concentration or **e** 100 ng/ml of pimecrolimus for 72 h. **a** The levels of PGE_2_ in the cell culture supernatant of hUCB-MSCs were determined using an ELISA. **B**–**c** The expression of the COX2 mRNA was determined using **b** conventional PCR and **c** Quantitative Real-time PCR. **d** Western blotting data and quantification showed the relative expression levels of COX2 in hUCB-MSCs. Expression of COX2 was normalized against GAPDH housekeeping gene by ImageJ program. **e** The expression of COX2 in hUCB-MSCs was verified by immunostaining, Bar = 500 μm. In vitro experiments were performed in triplicate using hUCB-MSCs isolated from each donor (*N* = 3). The Kruskal–Wallis test with Dunn’s post hoc was used to compare the pimecrolimus-treated hUCB-MSCs with control group. n.s: not significant, *P < 0.05, **P < 0.01 and ***P < 0.001, ****P < 0.0001. The results are presented as the means ± SD

Various cellular mechanisms are related to hUCB-MSC-mediated immunomodulation, and secretion of soluble factors plays a crucial role [25]. We performed ELISA assay to investigate whether pimecrolimus decreases the production of factors related to immunomodulation in AD, including IDO-1, TGF-beta, and PGE_2_. The production of IDO-1 by IFN-γ stimulation was not altered by pimecrolimus treatment (Additional file 3: Figure S3A). The secretion of TGF-beta from hUCB-MSCs was also not significantly different after pimecrolimus treatment (Additional file 3: Figure S3B). However, treatment with pimecrolimus reduced the secretion of PGE_2_ in a dose-dependent manner (Fig. 4a). PGE_2_ is one of the essential homeostatic factors that plays a key role in MSC-mediated immunomodulation. PGE_2_ is synthesized from arachidonic acid released from membrane phospholipids by cyclooxygenase (COX) [26]. In our previous studies, we identified that COX2-PGE_2_ axis as a crucial factor responsible for the immunosuppressive properties of hUCB-MSCs [11, 27]. Thus, we performed conventional and Quantitative Real-time PCR analysis, and found that pimecrolimus dose-dependently decreased COX2 mRNA expression level in hUCB-MSCs (Fig. 4b, c). The dose-dependent decrease in COX2 expression was also confirmed by Western blotting (Fig. 4d), and immunostaining analysis also revealed that the COX2 expression level was decreased by pimecrolimus treatment (Fig. 4e). Based on these findings, pimecrolimus decreased the production of PGE_2_ as the expression of COX2 was dose-dependently suppressed.

### Pimecrolimus downregulates the COX2-PGE_2_ axis by inhibiting the nuclear translocation of NFAT3 in hUCB-MSCs

**Fig. 5:**
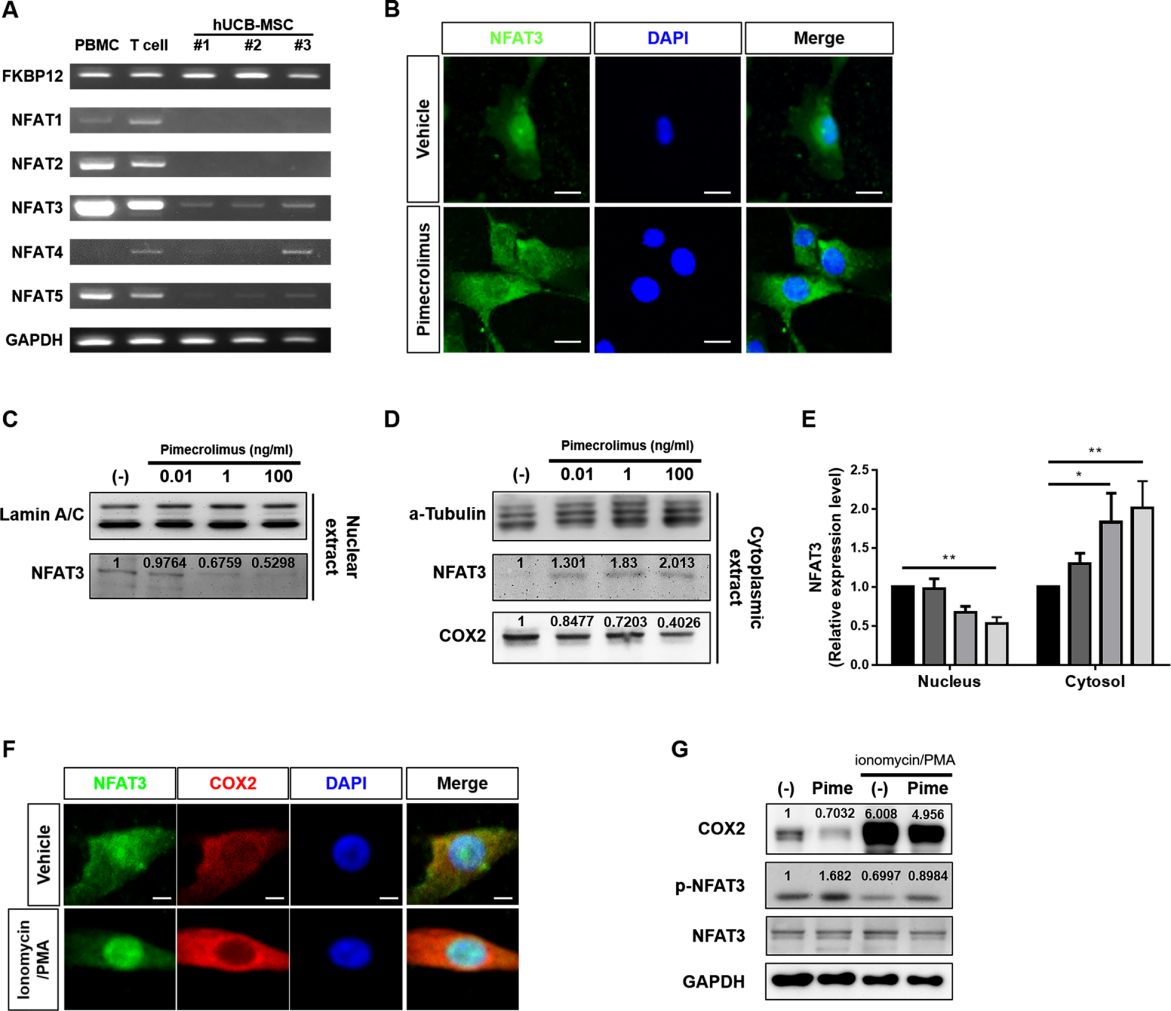
Pimecrolimus downregulates the COX2-PGE_2_ axis in hUCB-MSCs by inhibiting nuclear localization of NFAT3. **a** The mRNA expression of NFAT isoforms and FKBP1 was determined using conventional PCR. hPBMCs or T cells were used as positive controls. **b** The expression of the NFAT3 in hUCB-MSCs was verified by immunostaining, Bar = 200 μm. Cells were treated with pimecrolimus (100 ng/ml). **c**–**e** Nuclear (**c**) and Cytosolic (**d**) fractions were analyzed using Western blotting. hUCB-MSCs were treated with the indicated concentration of pimecrolimus for 3 days. The specificity of the nuclear and cytosolic extracts was determined by performing Western blot analysis with lamin A/C (nuclear) and α-tubulin (cytoplasmic) antibodies. Quantification of NFAT3 and cytosolic COX2 was performed by Image J, and normalized by lamin A/C (nuclear fraction) or α-tubulin (cytosolic fraction). The graph **e** represents the relative expression level of NFAT3 in nucleus and cytosol of hUCB-MSCs dependent on pimecrolimus treatment. **f** The expression of NFAT3 and COX2 in hUCB-MSCs. Cells were treated with ionomycin (1 μg/ml) and PMA (100 ng/ml) for 72 h. Bar = 200 μm. **g** Western blot analysis of hUCB-MSCs revealed COX2 expression and NFAT phosphorylation. Cells were treated with ionomycin (1 μg/ml) and PMA (100 ng/ml) for 72 h with or without pimecrolimus (100 ng/ml). Quantification of COX2 and phosphorylated NFAT3 was performed by imageJ. Expression of COX2 was normalized against GAPDH housekeeping gene, and expression of phosphorylated NFAT3 was normalized against NFAT3 and GAPDH housekeeping gene. The Kruskal–Wallis test with Dunn’s post hoc was used in (**e**). *P < 0.05 and **P < 0.01. In vitro experiments were performed in triplicate using hUCB-MSCs isolated from each donor (*N* = 3)

As mentioned above, pimecrolimus inhibits calcineurin and prevents the nuclear translocation of NFAT. NFAT proteins or their mRNAs have been detected outside the immune system, suggesting their possible involvement in the development or function of other systems [28]. Next, we hypothesized that pimecrolimus inhibits the COX2-PGE_2_ axis in hUCB-MSCs by regulating NFAT translocation. First, we screened which of the five members of the NFAT family were expressed in hUCB-MSCs. We performed a conventional RT-PCR analysis of the five NFAT family members to evaluate their mRNA expression levels in hUCB-MSCs (Fig. 5a). RNA extracted from hUCB-MSCs was analyzed in parallel with RNA from whole hPBMCs or isolated T cells from hPBMCs. The mRNA expression of FKBP12, a protein to which pimecrolimus binds that regulates NFAT activation, was confirmed in hUCB-MSCs. NFAT1 and NFAT2 mRNA expressions were not detected in hUCB-MSCs, whereas these mRNAs were expressed in hPBMCs and T cells, as previously described [29]. Although variations were observed between cell lines, the expression of NFAT3, NFAT4 and NFAT5 was detected in hUCB-MSCs. We focused on NFAT3 because its expression was confirmed in all 3 lines of hUCB-MSCs. Next, we investigated whether pimecrolimus blocked the nuclear translocation of NFAT3. In the vehicle group, NFAT3 was detected in both the nucleus and cytosol, but it was predominantly located in the nuclear fraction (Fig. 5b). Interestingly, NFAT3 was mostly located in the cytosolic fraction of hUCB-MSCs after pimecrolimus treatment. Western blot analysis of subcellular fractions from hUCB-MSCs also showed that the expression of NFAT3 in the nuclear localization decreased dose-dependently by pimecrolimus treatment (Fig. 5c, e). The expression of NFAT3 in the cytosolic fraction, conversely, increased by pimecrolimus (Fig. 5d, e). A subsequent decrease in COX2 levels in cytosolic fraction was observed (Fig. 5d). Coactivation of cells with ionomycin and phorbol myristate acetate (PMA) is known to result in a significant upregulation of NFAT-dependent gene expression by inducing the dephosphorylation of NFAT. Next, hUCB-MSCs were treated with ionomycin/PMA. Although NFAT3 was detected in both the cytosol and nucleus in resting hUCB-MSCs, ionomycin/PMA accelerated the nuclear translocation of NFAT3 and increased COX2 expression (Fig. 5f). Thus, COX2 expression in hUCB-MSCs depended on NFAT3 signaling. Additionally, by performing a Western blot analysis, we confirmed that the level of COX2 was increased by treatment with ionomycin/PMA, and the level of COX2 in pimecrolimus-treated hUCB-MSCs was decreased under both resting and ionomycin/PMA-treated conditions (Fig. 5g). These results suggested that NFAT activation by ionomycin/PMA increased COX2 expression and that pimecrolimus inhibited COX2 expression. We further analyzed the phosphorylation of NFAT3. NFAT must be dephosphorylated for activation in the nucleus, and thus phosphorylated NFAT indicates a cytosolic localization. After treatment with pimecrolimus, the level of phosphorylated NFAT3 was increased. When hUCB-MSCs were treated with ionomycin/PMA, the level of phosphorylated NFAT3 was decreased, indicating a reduction in the cytosolic localization of NFAT3. However, after treatment with pimecrolimus, the level of phosphorylated NFAT3 was increased in both resting and ionomycin/PMA-treated hUCB-MSCs. Taken together, these observations indicate that NFAT3 is one of the crucial transcription factors in hUCB-MSCs that induced COX2 expression and pimecrolimus administration interfered with its function.

## Discussion

AD is a chronic and inflammatory cutaneous disease that results from complex genetic, epigenetic, environmental and immunological interactions with an overlapping epidermal barrier defect [30]. The treatment of AD is based on emollients, anti-inflammatory drugs, antihistamines and antibiotics. Many patients have already been prescribed to use drugs in combination. Combination therapy is particularly beneficial when it provides additive or synergistic efficacy, often reducing the required doses of each drug compared with monotherapy and potentially limiting side effects. However, not all drug combinations work well, and may lead to the inactivation of other drugs or an exacerbation of side effects in some cases. Therefore, the combination of drugs should be confirmed to exert complementary effects rather than antagonistic or redundant effects [31]. In terms of mesenchymal stem cells, several studies suggest enhancement strategies via combination therapy with other agents. For example, coadministration with graphene oxide flakes or the immunomodulatory microparticle MIS416, has been reported to enhance the implantation of MSCs into the lesion [32, 33]. Although many studies have focused on resolving the limitations of hMSCs therapy, however researchers have not reported whether a combination method disrupts the therapeutic potential of mesenchymal stem cells. Therefore, we focused on the interaction of hUCB-MSCs and a topical calcineurin inhibitor, one of the most common anti-inflammatory drugs used to treat AD.

In this study, coadministration with pimecrolimus reduced the therapeutic effects of hUCB-MSCs on AD. Pimecrolimus cream (1%), a topical calcineurin inhibitor, has been used to treat AD. Pimecrolimus targets T cells and mast cells and inhibits the production and release of cytokines and other inflammatory mediators [34]. It binds with high affinity to FKBP12 and inhibits the activity of calcineurin, a calcium-dependent phosphatase. Calcineurin dephosphorylates NFAT, one of the most important transcription factors in T cells, and induces its translocation into the nucleus and activation. Therefore, pimecrolimus inhibits calcineurin-mediated NFAT activation in T cells, resulting a blockade of the transcription of inflammatory cytokines. Several studies on the effects of calcineurin inhibitors on the other cell types, in addition to T cells, have been reported [35–37]. NFAT transcription factors play a key role in T cell activation and differentiation. Recent studies have revealed that they also play an important role in other immune cell types, including dendritic cells, mast cells, B cells, natural killer T cells and megakaryocytes [38–40]. NFAT proteins are also involved in various developmental processes, including those of the heart, skeletal muscle, smooth muscle, vasculature, neurons, bone, pancreas and skin [41]. The NFAT family consists of five proteins: NFAT1 (NFATc2), NFAT2 (NFATc1), NFAT3 (NFATc4), NFAT4 (NFATc3), and NFAT5 [42]. NFAT1 and NFAT2 are the major NFATs involved in T cell activation, NFAT3 is expressed primarily in nonlymphoid tissues, NFAT4 is expressed mainly in the thymus, and NFAT5 is a transcription factor crucial for cellular response to hypertonic stress [28]. We first identified the expression of the NFAT family and FKBP12 in hUCB-MSCs. Among the 5 NFAT family members, only NFAT3 was expressed in all 3 hUCB-MSC lines, and NFAT4 was also expressed in one cell line. Notably, NFAT3 proteins are partially constitutively active in hUCB-MSCs, even in resting cells, as judged by their subcellular localization. This result suggested that NFAT3 may mediate the transcription of some genes in hUCB-MSCs.

We observed that pimecrolimus treatment inhibited the nuclear translocation of NFAT3 and downregulated the expression of COX2, suggesting an involvement of NFAT in the transcription of the COX2 gene. The human COX2 promoter contains binding sites for NF-κB, NF-IL6, CRE binding proteins, and NFAT [43]. The induction of COX2 expression by NFAT has been reported in human T cells, breast cancer cells, keratinocytes and vascular smooth muscle cells [43–46]. However, our study is the first to identify the induction of COX2 expression by NFAT in human mesenchymal stem cells, and more importantly, we showed that the combination of pimecrolimus and hMSC therapy potentially leads to an antagonized result.

hMSCs have been known to exert immunomodulatory functions via several soluble factors including TGF-beta, HGF, IL-6, IL-10, NO and PGE_2_ [47]. Among these factors, PGE_2_ is an especially important soluble factor that attenuates the crucial phenotype of AD progression. It is known to regulate many immune cells associated with AD, including T cells, monocytes, B cells and NK cells [48]. Thus, COX2, a key enzyme involved in the production of PGE_2,_ is also considered a crucial indicator of the immunomodulatory efficacy of hUCB-MSCs [49]. Shin et al. and Lee et al. reported that hMSCs exert inhibitory effects on IgE production by B cells, but these effects were attenuated by celecoxib, a selective COX2 inhibitor. Inhibition of mast cell degranulation by hMSCs has also been reported to be mediated by COX2-PGE_2_ pathway, and hMSCs treated with celecoxib exert a weak inhibitory effect on mast cell degranulation [12, 16]. Additionally, in our study, pimecrolimus downregulated the expression level of COX2 in hUCB-MSC, and hUCB-MSCs treatment with pimecrolimus did not induce immunosuppression against Th2 cells and mast cells than control group. Taken together, these results imply that the COX2-PGE_2_ axis is critical for hUCB-MSCs to exert their immunosuppressive function against AD.

The present study revealed that coadministration with pimecrolimus hindered the therapeutic effects of hUCB-MSCs on AD by inhibiting COX2 expression via the NFAT-COX2-PGE_2_ axis. As the therapeutic use of human mesenchymal stem cells has become more widespread, interactions with other drugs should be elucidated for safety and efficacy issues. We first documented that pimecrolimus influenced the effectiveness of hMSC therapy in AD in animal models and in vitro. Thus, from the perspective of hMSC therapy, we suggest that calcineurin inhibitors, especially pimecrolimus, should be used under appropriate medical oversight. This finding could be a vital factor behind the unsatisfactory results in clinical trials of hMSC therapy. For more efficient and definite curative effects, an adjustment of medication period might be suggested as a solution. Furthermore, current mainstay treatments for AD include a large variety of drugs, such as topical corticosteroids, antihistamines or antibiotics. In our previous study, histamine played a role in the COX2-mediated immunomodulatory ability of hUCB-MSCs through the activation of its receptors H1 and H4 [16]. This result implied that coadministration of antihistamine and hUCB-MSC therapy might affect the therapeutic outcome. Therefore, studies elucidating the interaction with other drugs and hMSCs might facilitate the improvement of clinical applications and expand our knowledge regarding the mechanisms of hMSC therapy for AD.

## Conclusions

hMSCs therapy holds considerable promise as an alternative therapeutic reagent for allergic disorders including AD. However, it is unclear whether combination strategy with other drugs might affect the result of cellular therapy. In the current study, we identified that pimecrolimus, one of the most common prescriptions for AD, could suppress the therapeutic potential of hMSC therapy. Further, we identified that pimecrolimus regulates the nuclear translocation of NFAT, and NFAT acts as a transcription factor for COX2 which is a crucial factor of immunomodulation of hMSCs. Our approach for combination therapy offers possibilities that simultaneous use of other drugs with hMSCs could influence the therapeutic outcomes of cell therapy and provides the improvement of the clinical application.

## Supplementary Information

**Additional file 1**.** Figure S1**: Coadministration of hUCB-MSCs and pimecrolimus inhibits the regulatory effect on atopic dermatitis in ear lesions. (A) Representative images of H&E-stained sections of ear lesions, Bar = 100 μm. (B) The thickness of the ear was measured in H&E-stained sections. (C-D) Expression levels of the TARC (C) and IL-22 (D) mRNAs in ear lesions were determined using Quantitative Real-time PCR. NC: negative control group, PC: positive control group (Df-induced group), Pimecrolimus: Df-induction + pimecrolimus, MSC: Df-induction + hUCB-MSCs, MSC+Pime.: Df-induction + hUCB-MSCs + pimecrolimus. The Kruskal-Wallis test with Dunn’s post hoc test was used to compare other groups with the Df-induced group (B-D). N = 3−5 mice per group. n.s: not significant and *P<0.05. The results are presented as the means ± SD.

**Additional file 2**.** Figure S2**: Pimecrolimus does not alter the fundamental properties of hUCB-MSCs. hUCB-MSCs were treated with the indicated concentrations of pimecrolimus for 3 days, and further analyses were conducted. (A) Analysis of cell viability using the MTT assay. (B) Representative bright-field microscopy images of hUCB-MSCs, Bar = 500 μm. (C) The proliferation of hUCB-MSCs was determined using the BrdU assay. (D) Cell cycle assay. (E) Cell surface marker expression on hUCB-MSCs. The expression profile of cell surface markers in hUCB-MSCs was measured using flow cytometry analysis. Negative markers: CD45, CD34 and CD36. Positive markers: CD105, CD29 and CD73. Histograms show a representative result. In vitro experiments were performed in triplicate using hUCB-MSCs isolated from each different donor (N=3). The Kruskal-Wallis test with Dunn’s post hoc test was used to compare treated cells with nontreated cells. (A-B). n.s: not significant and ****P<0.0001. The results are presented as the means ± SD.

**Additional file 3**.** Figure S3**: Pimecrolimus does not influence the production of TGF-beta and IDO-1 from hUCB-MSCs. hUCB-MSCs were treated with indicated concentration of pimecrolimus for 3 days. (A) To measure the production of IDO-1 in hUCB-MSCs, cells were treated with pimecrolimus and IFN-γ (20 ng/ml). The levels of IDO-1 (cell lysates) from hUCB-MSCs were determined by ELISA. (B) After pimecrolimus treatment, the secretion of TGF-beta (cell supernatant) from hUCB-MSCs was determined by ELISA. In vitro experiments were performed in triplicate using hUCB-MSCs isolated from each different donor (N=3). All experiments in this figure were analyzed using the Kruskal-Wallis test with Dunn’s post hoc test. n.s: not significant. Results are shown as the mean ± SD.

**Additional file 4. Table S1**: Primer sequences used in this paper.

## Data Availability

The datasets used and/or analyzed during the current study are available from the corresponding author on reasonable request.

## Abbreviations

hMSCs: Human mesenchymal stem cells
AD: Atopic dermatitis
hUCB-MSCs: Human umbilical cord blood mesenchymal stem cells
Th2 cell: Type 2 helper T cell
FKBP-12: FK506-binding protein-12
NFAT: Nuclear factor of activated T cells
MSC: Mesenchymal stem cell
PGE_2_: Prostaglandin E2
COX2: Cyclooxygenase 2
Df: Dermatophagoides farinae
H&E: Hematoxylin and eosin
RhSCF: Recombinant human stem cell factor
BrdU: Bromodeoxyuridine
hPBMCs: Human peripheral blood mononuclear cells
COX: Cyclooxygenase
PMA: Phorbol myristate acetate
