# Supplementary figures and images for: Pimecrolimus interferes the therapeutic efficacy of human mesenchymal stem cells in atopic dermatitis by regulating NFAT-COX2 signaling
Source: Stem Cell Res Ther. 2021 Aug 28;12:482. doi: 10.1186/s13287-021-02547-8 (PMC8399851; doi:10.1186/s13287-021-02547-8)

**A**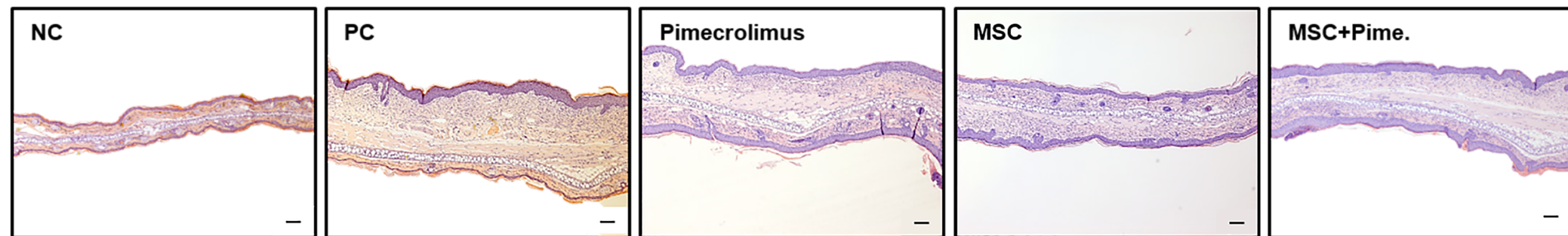**B**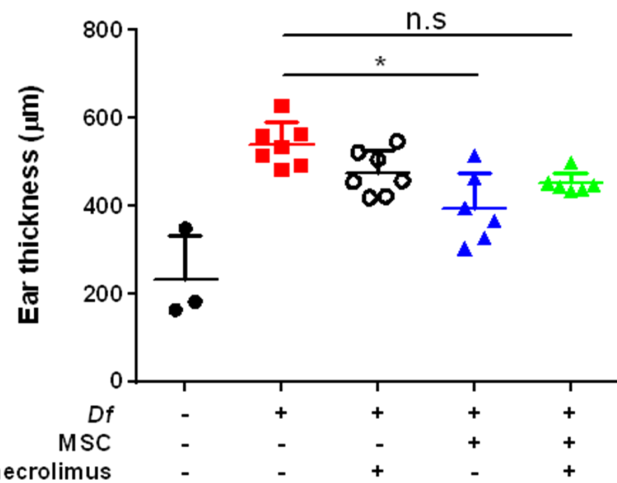**C**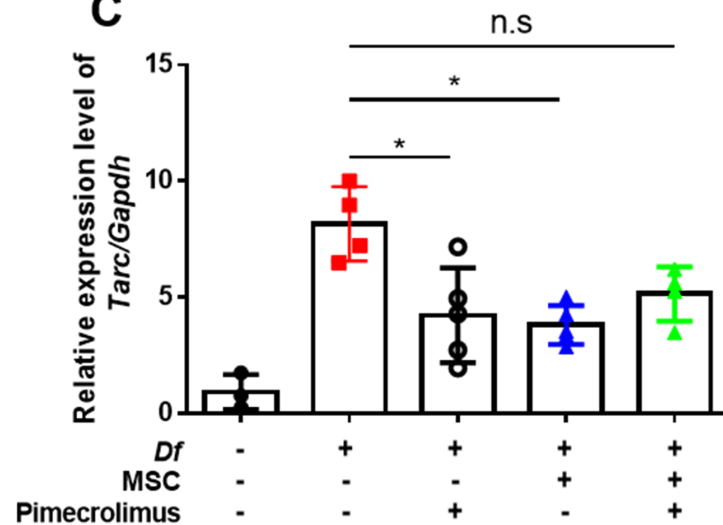**D**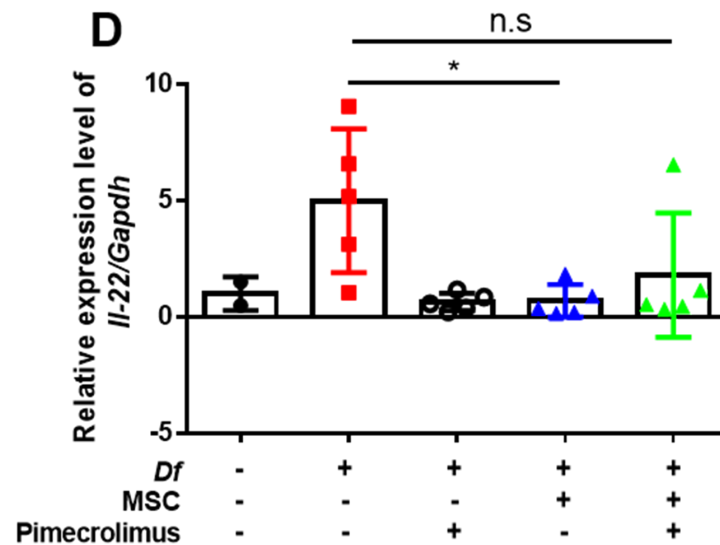

Supplement: Supplementary file 1 — Additional file 1. Figure S1: Coadministration of hUCB-MSCs and pimecrolimus inhibits the regulatory effect on atopic dermatitis in ear lesions. (A) Representative images of H&E-stained sections of ear lesions, Bar = 100 μm. (B) The thickness of the ear was measured in H&E-stained sections. (C-D) Expression levels of the TARC (C) and IL-22 (D) mRNAs in ear lesions were determined using Quantitative Real-time PCR. NC: negative control group, PC: positive control group (Df-induced group), Pimecrolimus: Df-induction + pimecrolimus, MSC: Df-induction + hUCB-MSCs, MSC+Pime.: Df-induction + hUCB-MSCs + pimecrolimus. The Kruskal-Wallis test with Dunn’s post hoc test was used to compare other groups with the Df-induced group (B-D). N = 3−5 mice per group. n.s: not significant and *P<0.05. The results are presented as the means ± SD. [file 13287_2021_2547_MOESM1_ESM.pdf]

**A**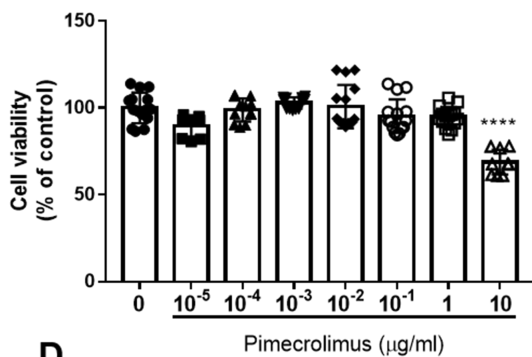**B**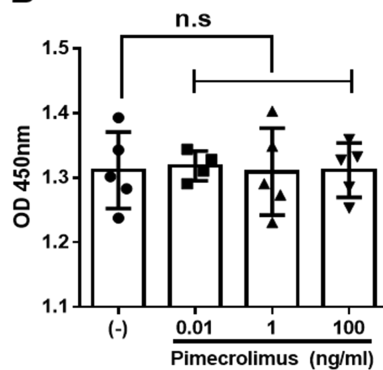**C**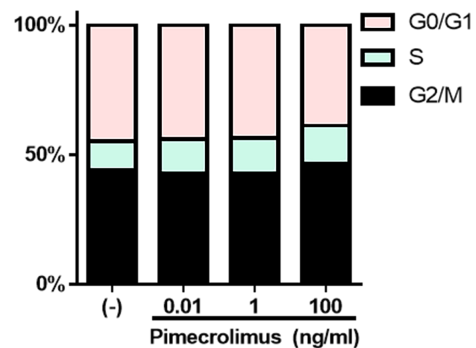**D**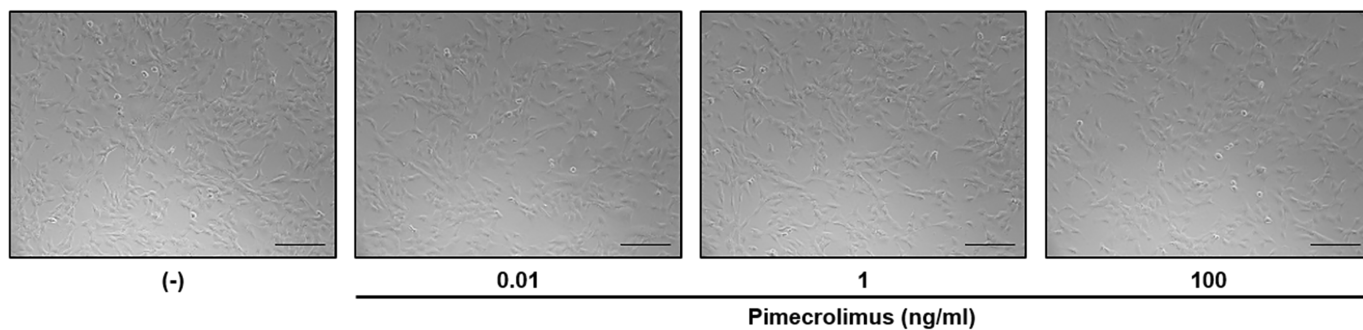**E**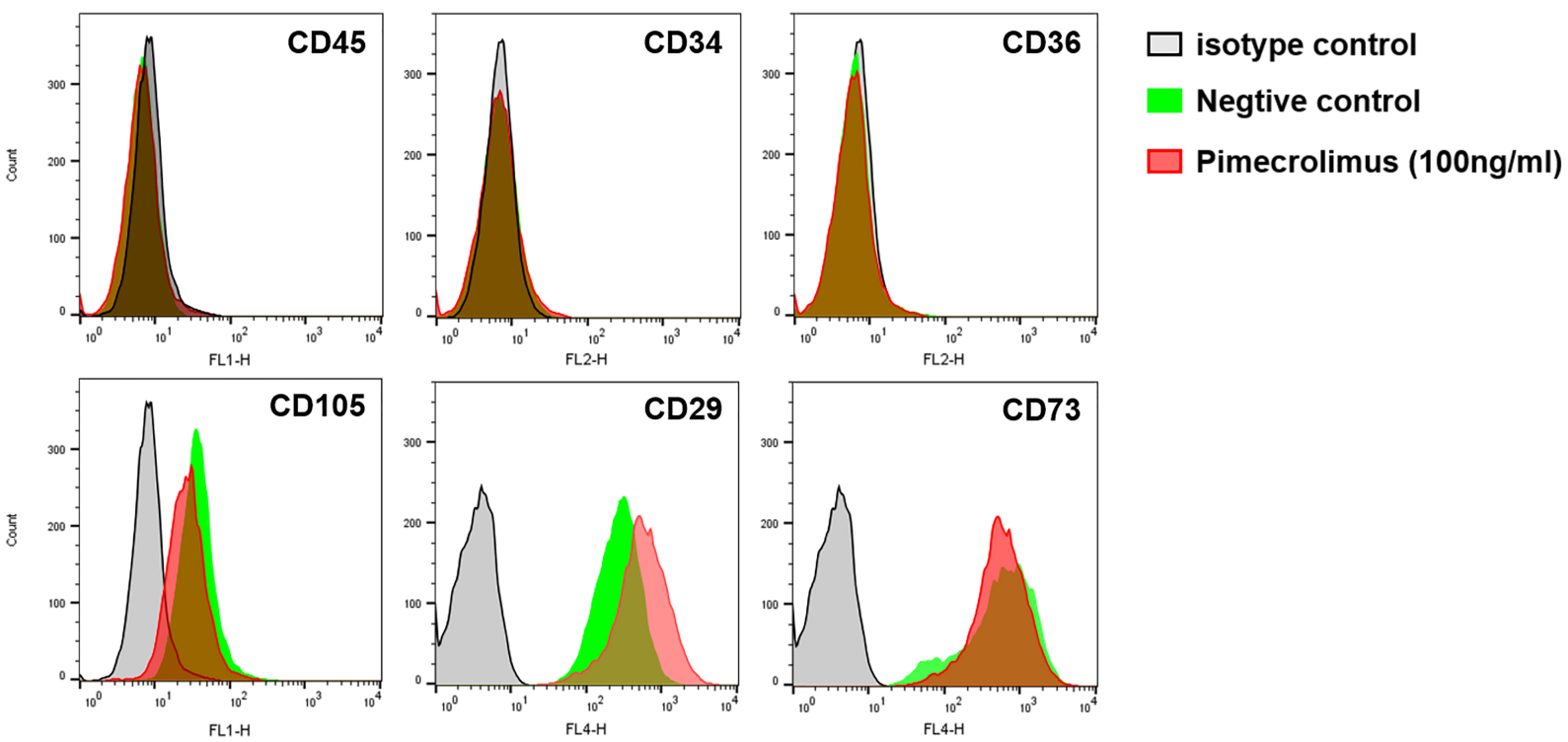

Supplement: Supplementary file 2 — Additional file 2. Figure S2: Pimecrolimus does not alter the fundamental properties of hUCB-MSCs. hUCB-MSCs were treated with the indicated concentrations of pimecrolimus for 3 days, and further analyses were conducted. (A) Analysis of cell viability using the MTT assay. (B) Representative bright-field microscopy images of hUCB-MSCs, Bar = 500 μm. (C) The proliferation of hUCB-MSCs was determined using the BrdU assay. (D) Cell cycle assay. (E) Cell surface marker expression on hUCB-MSCs. The expression profile of cell surface markers in hUCB-MSCs was measured using flow cytometry analysis. Negative markers: CD45, CD34 and CD36. Positive markers: CD105, CD29 and CD73. Histograms show a representative result. In vitro experiments were performed in triplicate using hUCB-MSCs isolated from each different donor (N=3). The Kruskal-Wallis test with Dunn’s post hoc test was used to compare treated cells with nontreated cells. (A-B). n.s: not significant and ****P<0.0001. The results are presented as the means ± SD. [file 13287_2021_2547_MOESM2_ESM.pdf]

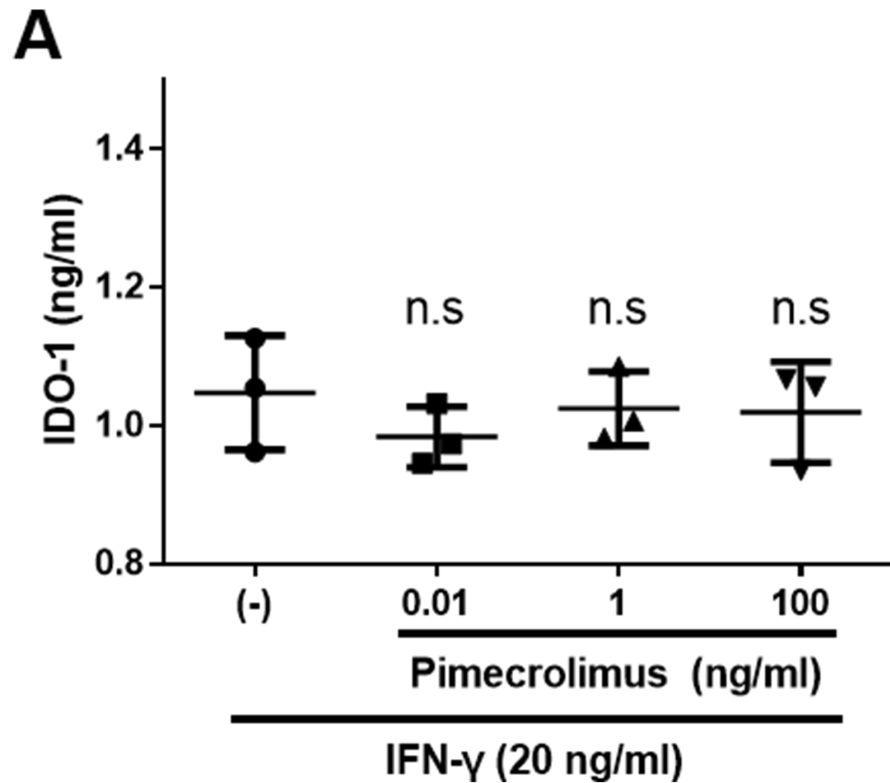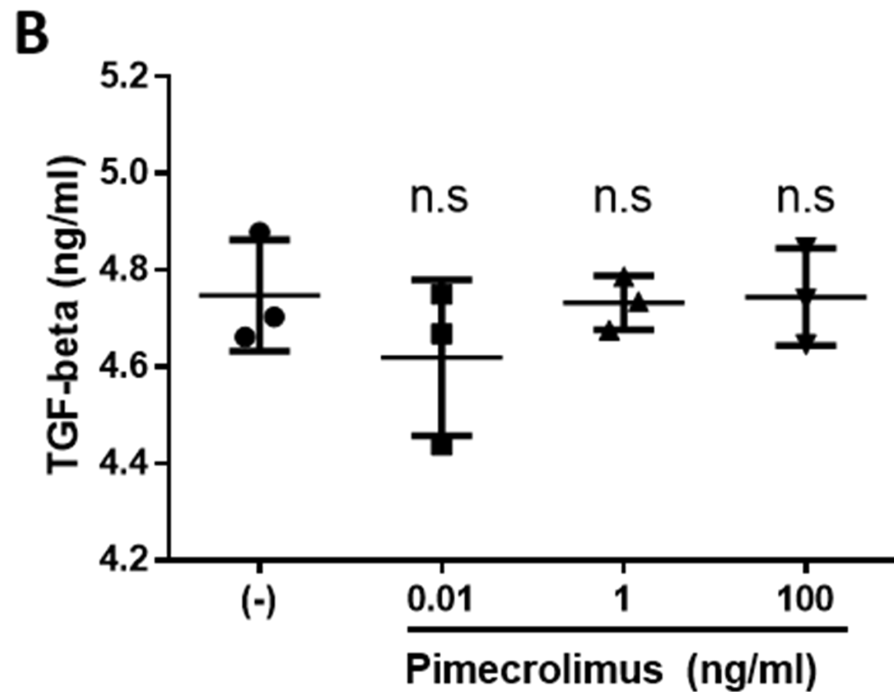

Supplement: Supplementary file 3 — Additional file 3. Figure S3: Pimecrolimus does not influence the production of TGF-beta and IDO-1 from hUCB-MSCs. hUCB-MSCs were treated with indicated concentration of pimecrolimus for 3 days. (A) To measure the production of IDO-1 in hUCB-MSCs, cells were treated with pimecrolimus and IFN-γ (20 ng/ml). The levels of IDO-1 (cell lysates) from hUCB-MSCs were determined by ELISA. (B) After pimecrolimus treatment, the secretion of TGF-beta (cell supernatant) from hUCB-MSCs was determined by ELISA. In vitro experiments were performed in triplicate using hUCB-MSCs isolated from each different donor (N=3). All experiments in this figure were analyzed using the Kruskal-Wallis test with Dunn’s post hoc test. n.s: not significant. Results are shown as the mean ± SD. [file 13287_2021_2547_MOESM3_ESM.pdf]
