# Supplementary material for: Pimecrolimus interferes the therapeutic efficacy of human mesenchymal stem cells in atopic dermatitis by regulating NFAT-COX2 signaling
Source: Stem Cell Res Ther. 2021 Aug 28;12:482. doi: 10.1186/s13287-021-02547-8 (PMC8399851; doi:10.1186/s13287-021-02547-8)
Supplement: Supplementary file 4 — Additional file 4. Table S1: Primer sequences used in this paper. [file 13287_2021_2547_MOESM4_ESM.pdf]

Supplementary Table 1. Primer sequences used in this paper.

Conventional RT-PCR					
**Gene name**		**Primer sequences**	**Annealing temperature (℃)**	**Cycles**	**Product length (bp)**
**hCOX2**	Forward	GGC CAT GGG GTG GAC TTA AA	60	35	181
	Reverse	ACC GTA GAT GCT CAG GGA CT			
**hNFAT1**	Forward	AAA CTC GGC TCC AGA ATC CA	60	35	578
	Reverse	TGG ACT CTG GGA TGT GAA CT			
**hNFAT2**	Forward	GCT ATG CAT CCT CCA ACG TC	60	35	441
	Reverse	AGT TGG ACT CGT AGG AGG AG			
**hNFAT3**	Forward	ACA CAG CCC TAT CTT CAG GA	60	35	294
	Reverse	ATC TTG CCT GTG ATA CGG TG			
**hNFAT4**	Forward	ACC CTT TAC CTG GAG CAA AC	60	35	339
	Reverse	CTT GCA GTA GCG ACT GTC TT			
**hNFAT5**	Forward	CGT GTG TGT GGC TTC TAT GT	60	35	329
	Reverse	TGC CTC TCA ATC AGA GAG AG			
**hFKBP12**	Forward	TGC TAG GCA AGC AGG AGG TGA T	60	35	109
	Reverse	GTG GCA CCA TAG GCA TAA TCT GG			
**hGAPDH**	Forward	TGA TGA CAT CAA GAA GGT GGT G	60	24	207
	Reverse	ACC CTG TTG CTG TAG CCA AAT			

Quantitative Real-time PCR				
**Gene name**		**Primer sequences**	**Annealing temperature (℃)**	**Product length (bp)**
**mTARC**	Forward	CAA GCT CAT CTG TGC AGA CC	60	219
	Reverse	CGC CTG TAG TGC ATA AGA GTC C		
**mIL-22**	Forward	TCA GTG CTA AGG ATC AGT GCT	60	136
	Reverse	TGA TTG CTG AGT TTG GTC AGG		
**mGAPDH**	Forward	GGC ATT GCT CTC AAT GAC AA	60	200
	Reverse	TGT GAG GGA GAT GCT CAG TG		
**hGATA3**	Forward	CAT GAA GCT GGA GTC GTC CC	60	344
	Reverse	GCC TTC GCT TGG GCT TAA TG		
**hSTAT6**	Forward	GAC ACT GTC CAG CAC CTT CA	60	190
	Reverse	GGA AAG GCA TTG GCA AGT GG		
**hIL-5**	Forward	AAC TGT GCA AGG GGG TAC TG	60	103
	Reverse	TCT CCG TCT TTC TTC TCC ACA C		
**hIL-13**	Forward	ATG GCG CTT TTG TTG ACC AC	60	180
	Reverse	AGC TGT CAG GTT GAT GCT CC		
**hCOX2**	Forward	GGC CAT GGG GTG GAC TTA AA	60	181
	Reverse	ACC GTA GAT GCT CAG GGA CT		
**hGAPDH**	Forward	TGA TGA CAT CAA GAA GGT GGT G	60	207
	Reverse	ACC CTG TTG CTG TAG CCA AAT		
